# Measles outbreak in a poorly vaccinated region in Cameroon: a case series study, public health challenges and recommendations

**DOI:** 10.11604/pamj.2015.22.163.8015

**Published:** 2015-10-20

**Authors:** Tsi Njim, Leopold Ndemnge Aminde, Fambombi Vitalis Feteh, Joel Mbigha Ngum, Chandini Aliyou Moustapha

**Affiliations:** 1 Regional Hospital Bamenda, North west region, Bamenda, Cameroon; 2 Health and Human Development (2HD) Research Group, Douala, Cameroon; 3 Sub-divisional Hospital Nguti, South west region, Nguti, Cameroon; 4 Clinical Research Education, Networking and Consultancy (CRENC), Douala, Littoral, Cameroon; 5 Mbopi Baptist Hospital Douala, Douala, Cameroon; 6 Fundong District Hospital, North west region, Fundong, Cameroon; 7 Sub-divisional Hospital Misaje, North west region, Misaje, Cameroon

**Keywords:** Measles, outbreaks, Cameroon

## Abstract

Measles is a highly contagious viral infection and still a leading cause of vaccine-preventable deaths in Africa; especially in unvaccinated populations. We reviewed the medical reports of the measles outbreak that occurred in Misaje, in the North west region of Cameroon from 11/03/2015 to 14/05/2015. Six measles cases were recorded during this period; three of them complicated by bacterial infections. Measles should be considered as a differential diagnosis for any febrile rash especially among poorly vaccinated populations. Primary preventive methods implemented by clinicians could help control outbreaks; especially with delays in public health intervention. Also, gaps in health policies in Cameroon should be addressed to scale up vaccination coverage in remote communities like Misaje to reduce the incidence of measles outbreaks.

## Introduction

Measles (rubeola) is a highly contagious viral disease with a 90% secondary infection rate and significant morbidity and mortality in children and pregnant women [1]. It is estimated that measles is responsible for 8% of deaths from vaccine-preventable diseases in the world [2]. Due to its lethal nature, the World Health Organisation (WHO) advocates for more efforts towards the control of this disease [2]. These efforts should include: routine vaccination; supplementary immunisation activities (SIAs) and active surveillance [2–5]. In Cameroon, improved routine vaccination at 9 months of age (using a combined measles, rubella and mumps vaccine) and careful active surveillance (using Focal persons (FPs) trained in regular and active surveillance of the disease in health facilities), has led to some success in measles control. A 90% vaccination coverage has led to a decrease in annual incidence from 41 cases per 100,000 children in 2001-2004 to 2 cases per 100,000 children from 2005-2008 [4]. However, some health districts still have a low vaccination coverage, with a reported 78% in Misaje (a health area in the Nkambe health district, Northwest region of Cameroon) in 2014 [6]. This represents a problem as over 95% of a population has to be immunised in order to eliminate measles [7]. In this case series study, we report 6 cases of measles and point out some flaws in public health policies that need to be addressed to help in the control of this disease.

## Methods

A measles outbreak occurred during the period of March-May 2015 in Misaje, a village with a population of over 16,000 inhabitants. Yearly routine vaccination in Misaje from 2008 ranges from 60-78%. There has been no outbreak of measles in this region since 2010. Therefore, the WHO definition of an outbreak is satisfied [8]. After suspicion of any case, socio-demographic data; clinical information including the date of appearance of the rash and other symptoms were recorded. Blood samples were collected from all the cases and sent to a reference laboratory; Centre Pasteur du Cameroun (CPC) in the capital city: Yaoundé, for detection of Ig-M antibodies for confirmation of measles. After the outbreak, all this information was later collected after revisiting the consultation registers and the public health reports written by the public health authorities [2].

## Results

A total of 6 measles cases were diagnosed and investigated (Table 1)

**Table 1:** Socio-demographic and clinical parameters of cases during the measles outbreak which occurred in Misaje from 11^th^ March to 14^th^ April 2015

						Date of							
Case Number	Sex	Age	Case type	Vaccination status	Onset of illness	Appearance of rash	consultation	Fever	conjunctivitis	cough	coryza	Ig M antibodies	Outcome
1	F	10	PC	M	08/03	11/04	11/04	Y	Y	Y	Y	Y	A
2	M	4	SC	N	04/04	09/04	14/04	Y	Y	Y	Y	UN	A
3	M	3	SC	N	05/04	09/04	14/04	Y	Y	Y	Y	UN	A
4	F	2	SC	N	04/04	09/04	14/04	Y	Y	Y	Y	UN	A
5	F	3	SC	N	26/04	29/04	30/04	Y	Y	Y	Y	UN	A
6	F	1	SC	N	28/04	01/05	30/04	Y	Y	Y	Y	UN	A

PC: Primary case; SC: Secondary case; M: May have been vaccinated during mass campaign of 2006; N: No; Y: Yes; UN: Unknown; A: Alive

### Case 1 (Primary case)

A 10 year old girl was brought to hospital with a 3 day history of a fever (temperature 40.5^°^C), cough, coryza and conjunctivitis on 11/03/2015. The hospital focal person (FP) screened her and informed the attending physician on a suspicion of measles. Activities during the previous 14 days included: fetching water from the local stream. On examination, she had normal anthropometric parameters, a generalised maculopapular erythematous rash and signs of right pulmonary consolidation. A provisional diagnosis of measles complicated by pneumonia was made. She was placed on intravenous fluids, broad spectrum antibiotics and antipyretics.

### Cases 2, 3 and 4

Patients 2 (4 years), 3 (3 years) and 4 (2 years) were siblings. They all presented with a 5 day history of a generalised papular erythematous rash (Temperatures >40.3^°^C), conjunctivitis, cough and coryza on 14/04/2015. Patient 2 had turbid urine for which a urinalysis showed proteins, leucocytes and nitrites. A provisional diagnosis of measles complicated by a urinary tract infection was made. Patient 3 had additional symptoms of bloody watery stools and vomiting which was suggestive of a bacillary dysentery. Patient 2 and 3 were placed on appropriate antibiotherapy. Patients were placed on supportive therapy.

### Case 5 and 6

Both cases (1 and 4 years old respectively) presented on the 30/04/2015. They had a history of a fever, generalised rash, coryza, cough and conjunctivitis. They were admitted with a provisional diagnosis of measles and managed with supportive therapy. None of the children had received any measles-containing vaccine (MCV). All patients lived within a 3 km radius. All patients recovered and were advised to stay at home for 14 days after discharge.

### Public health intervention

**Figure 1:**
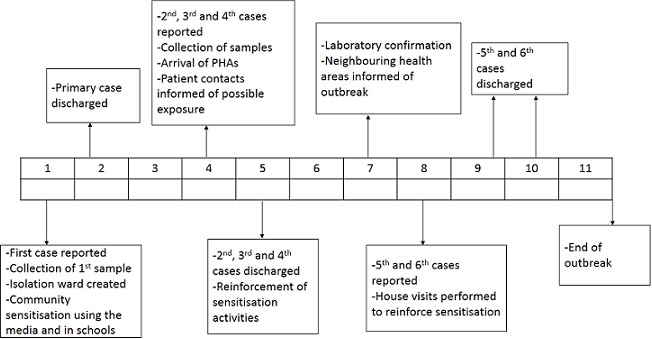
Weekly activities performed by the clinicians and the PHAs during the measles outbreak in Misaje from 11/03/2015 to 14/05/2015

Upon diagnosis, the children were isolated in a makeshift isolation ward. After collection of the samples, public health authorities (PHAs) were informed of a possible outbreak on 11/03/2015. Before laboratory confirmation of the outbreak and public health intervention, further preventive measures to control the spread were undertaken: individuals were prevented from visiting the isolation ward; information was spread over the local community radio and schools on the symptoms, methods of transmission, and the need for early arrival to the hospital upon recognition of symptoms. Also, contacts of the patients were informed of possible exposure. PHAs arrived on the field 3 weeks into the outbreak. They performed reviews of consultation registers and visited the homes of suspected cases as they attempted to establish limits of zones affected. Laboratory confirmation from CPC was received 6 weeks after the first case was reported. Health care providers (HCPs) and FPs were reminded on the recognition and management of measles. Neighbouring health areas were also informed on the outbreak and HCPs in these regions were informed to be on high alert. The outbreak lasted from the 11/03/2015 to 14/05/2015 with 6 cases reported in Misaje (Figure 1).

## Discussion

Measles is highly sensitive to vaccination programs [5]. Routine vaccination, supplementary immunization activities (SIAs) and active disease surveillance have shown to adequately decrease the frequency and severity of outbreaks [3–5]. The recent outbreak in Misaje pointed out flaws in the above policies aimed at controlling measles spread in Cameroon. Firstly, routine vaccination is highly inadequate as only a 78% coverage was achieved in this region in 2014 [6]. This represents a significant problem as over 95% of community members have to be vaccinated to achieve appropriate immunisation in this region [7]. Several children are missed because their parents complain of inaccessibility of the health structure. Also, this is a Fulani-dominated community and women cannot take children for vaccination without permission from the men. The general fear of vaccination and mistrust of the health system adversely contributes to the situation [4]. None of the cases in this study could provide confirmation of taking a MCV. There is therefore an urgent need for community education on the importance of vaccinations especially in indigenous populations.

Secondly, there has been no SIA in this region in the past 9 years. Also, a second dose of the measles vaccine is not provided in the Extended Program of Immunisation in Cameroon. A highly immunised population can limit the spread of measles [3]; with no SIAs, comprehensive measles vaccination coverage cannot be attained increasing the likelihood of outbreaks. The last SIA was a nationwide vaccination activity in 2006. Even the primary case in this study may not have been vaccinated during this SIA despite the 90% coverage [4]. This calls for more regular and expansive SIA in vulnerable populations in Cameroon. Thirdly, there was a late response from the PHAs. They presented on the field 3 weeks into the outbreak. The samples collected had to be sent to Yaoundé which is approximately 549.5 kilometres form Misaje; partly accounting for a long delay before the outbreak could be confirmed. Also, confirmation could not be received for all the cases despite sample collection. This is because the present public health policy of Cameroon advocates that after confirmation of the primary case, other cases should be considered as measles cases without further confirmation. Furthermore, outbreak-response immunisation (ORI) recommended by WHO, and essential in reducing measles related mortality [4] was not performed during and after this outbreak. This decreases the likelihood of converting such poorly vaccinated regions to fully immunised populations.

In developing countries, measles outbreaks are rare due to sufficient vaccination. Outbreaks in such countries are due to either imported cases from or suspicious contact with international travellers from measles endemic regions [2, 9, 10]. In Cameroon however, outbreaks are usually due to poor vaccination coverage; like the outbreak which occurred in the far north of the country in 2008 [4]. The recent outbreak in Misaje could be blamed on this insufficient coverage especially as all the cases were indigenes of this region, ruling out the possibility of importation. Hence, there is a great need for revision of health policies in order to scale up vaccination coverage especially as measles is clearly still endemic in this region. Finally, Vitamin A supplementation has been shown to reduce measles-related morbidity and mortality in acute crises [6]. Vitamin A was not available in Misaje during the outbreak. Public health institutions need to upgrade health care facilities to better respond to unforeseen outbreaks both clinically and public health wise. However, the introduction of FPs for active disease surveillance can help in easily identifying measles cases as demonstrated in case 1. Active surveillance with the availability of posters listing symptoms and management options were critical in the HCPs advocating the diagnosis of measles in the presence of a febrile rash. Also, despite the late arrival of the PHAs, the role of the clinicians in isolation of suspected cases and population sensitization using the media was pivotal in reducing the spread and containing the disease as only 6 cases occurred in Misaje.

## Conclusion

Measles outbreaks in poorly vaccinated regions could be challenging; especially with gaps in health policies and cultural beliefs. Interventions for the control of this disease need to be modified and or intensified to include: better coverage during routine immunisation by improving accessibility to health structures; introduction of SIAs and ORI during outbreaks; reducing the response time of PHAs to outbreaks and upgrading the knowledge of already available FPs to improve disease surveillance.
